# Structural Divergence in *O*-GlcNAc Glycans Displayed on Epidermal Growth Factor-like Repeats of Mammalian Notch1

**DOI:** 10.3390/molecules23071745

**Published:** 2018-07-17

**Authors:** Mitsutaka Ogawa, Yuya Senoo, Kazutaka Ikeda, Hideyuki Takeuchi, Tetsuya Okajima

**Affiliations:** 1 Department of Molecular Biochemistry, Nagoya University Graduate School of Medicine, 65 Tsurumai, Showa-ku, Nagoya 466-8550, Japan; mitsutaka.ogawa@med.nagoya-u.ac.jp (M.O.); y.senoo@med.nagoya-u.ac.jp (Y.S.); kazutaka.ikeda@riken.jp (K.I.); htakeuchi@med.nagoya-u.ac.jp (H.T.); 2 RIKEN, Center for Integrative Medical Sciences, 1-7-22 SuehirO-cho, Tsurumi, Yokohama 230-0045, Japan

**Keywords:** *O*-GlcNAc, Notch1, EOGT, EGF domain, *O*-GlcNAc glycan

## Abstract

Extracellular *O*-GlcNAc is a novel class of modification catalyzed by epidermal growth factor-like (EGF)-domain specific *O*-GlcNAc transferase (EOGT). In mammals, EOGT is required for ligand-mediated Notch signaling for vascular development. Previous studies have revealed that *O*-GlcNAc in mammalian cultured cells is subject to subsequent glycosylation, which may impose additional layers of regulation. This study aimed to analyze the *O*-GlcNAc glycans of *Drosophila* EGF20 as model substrates and mouse Notch1 EGF repeats by mass-spectrometry. The analysis of *Drosophila* EGF20 expressed in HEK293T cells revealed that the majority of the proteins are modified with an elongated form of *O*-GlcNAc glycan comprising terminal galactose or sialic acid residues. In contrast, recombinant Notch1 EGF repeats isolated from HEK293T cells revealed structural divergence of *O*-GlcNAc glycans among the different EGF domains. Although the majority of Notch1 EGF2 and EGF20 domains contained the extended forms of the glycan, the *O*-GlcNAc in many other domains mostly existed as a monosaccharide irrespective of the exogenous EOGT expression. Our results raised a hypothesis that an array of *O*-GlcNAc monosaccharides may impact the structure and function of Notch receptors.

## 1. Introduction

Extracellular *O*-GlcNAc modification was initially found on the epidermal growth factor-like (EGF) domain of *Drosophila* Notch receptor [1]. Although *O*-GlcNAc modification by *O*-GlcNAc transferase (OGT) occurs in the nucleus, cytoplasm, and mitochondria [2], the extracellular *O*-GlcNAc modification is catalyzed by the endoplasmic reticulum (ER)-localized EGF-domain specific O-GlcNAc transferase (EOGT) [3,4,5,6]. Genetic inactivation of *Eogt* in mice led to abnormal angiogenesis [7] and in humans results in Adams-Oliver syndrome, a congenital disease [8,9,10]. Loss of *O*-GlcNAc in the Notch1 receptor decreased the binding ability of Notch1 to DLL1/4 ligand and reduced the DLL1/4 ligand-induced Notch signaling in mammals [7]. In contrast, the EOGT knockout fly lacks an apparent Notch mutant phenotype [3,11]. Instead, the *Eogt* shows genetic interaction with *dp* that encodes extracellular matrix protein Dumpy. These results suggest that the function of *O*-GlcNAc is distinct in flies and mammals.

**Figure 1:**
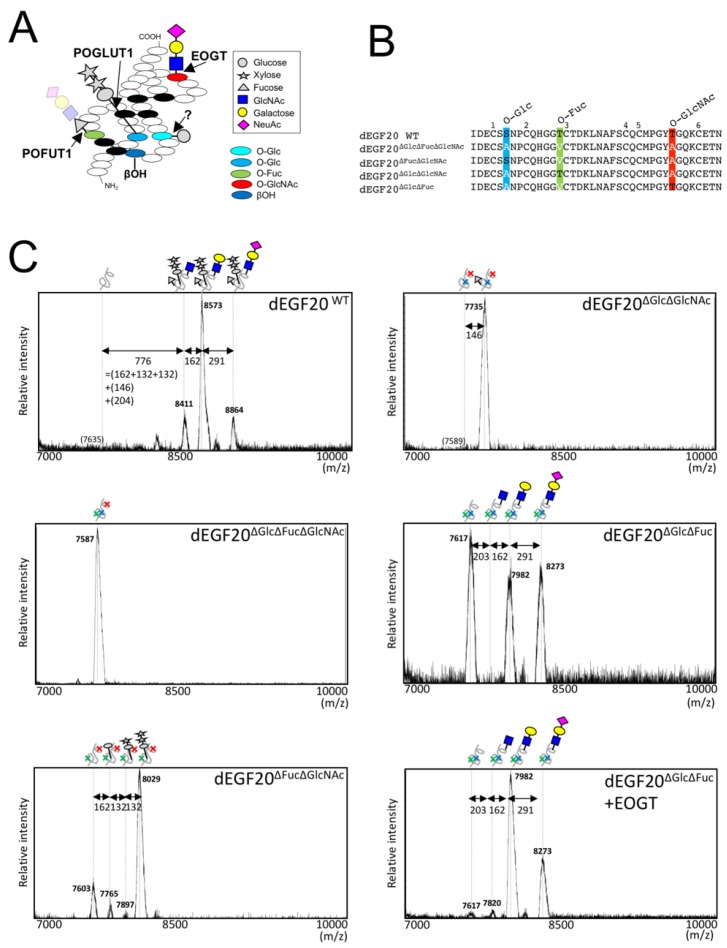
MALDI-TOF-MS analysis of Drosophila Notch EGF20 expressed in HEK293T cells. (**A**) The schematic diagram of EGF domains modified with the most extended forms of *O*-glycans. In HEK293T cells, *O*-Fuc modified by POFUT1 remains as a monosaccharide due to lack of Fringe activity responsible for elongation of *O*-Fuc. *O*-Glc modified by POGLUT1 can be elongated into a trisaccharide by the action of two distinct xylosyltransferases. Atypical *O*-Glc modification was found on Ser residue (*light blue*) of EGF11. Also shown are EOGT-catalyzed *O*-GlcNAc on Ser/Thr residue (*red*) and beta-hydroxylation (*dark blue*). (**B**) The amino acid sequence of wild-type *Drosophila* EGF20 (dEGF20^WT^) and its variants. Theoretical molecular weight (Mw) of dEGF20^WT^ is 7603.16; dEGF20^ΔGlcΔFucΔGlcNAc^, 7555.16; dEGF20^ΔFucΔGlcNAc^, 7571.16, dEGF20^ΔGlcΔGlcNAc^, 7557.13; dEGF20^ΔGlcΔFuc^, 7585.19. Modification sites for *O*-Glc (*blue*), *O*-Fuc (*green*), and *O*-GlcNAc (*red*) were highlighted. (**C**) Wild type dEGF20 and its variants were transiently expressed in HEK293T cells, isolated with anti-His antibody, and analyzed by MALDI-TOF-MS in a positive linear mode. Molecular mass of major peaks in each spectrum are shown. Peak-to-peak mass increments corresponding to glycosylation are indicated by *double-headed arrows*. Where indicated, EOGT is overexpressed in HEK293T cells. *Gray circle*, glucose; *gray star*, xylose; *yellow circle*, galactose; *blue square*, GlcNAc; *purple diamond*, NeuAc; *gray triangle*, fucose.

Three classes of atypical *O*-glycans and β-hydroxylation (βOH) occur in the EGF domains (Figure 1A). In mammals, *O*-linked fucose (*O*-Fuc) modified by POFUT1 occurs in the EGF domain at the position of C^2^XXXX(S/T)C^3^ (Figure 1B) [12,13]. *O*-Fuc is further modified with GlcNAc by Fringe [14], but the elongation of GlcNAc by Fringe does not occur in HEK293T cells [12]. *O*-glucose (*O*-Glc) catalyzed by POGLUT1 occurs in EGF domains at C^1^XSX(P/A)C^2^ [15,16], which could be further modified with two xylose (Xyl) residues, resulting in *O*-Glc-Xyl-Xyl structure by GXYLT1/2 [17] and XXYLT1 [18,19].

Another type of *O*-glucose is reported in the EGF11 domain (C^3^XXXXXSXXC^4^) [20,21]. Furthermore, β-hydroxylation (βOH) occurs in the EGF domain at C^3^X(D/N)XXXXXXC^4^ [22,23]. Finally, EOGT-catalyzed *O*-GlcNAcylation occurs in the EGF domain at C^5^XXXX(T/S)GXXC^6^ [7,24]. To date, the *O*-GlcNAc structure in the Notch receptor has been reported in *Drosophila* and mammals. In S2 cells, the extracellular *O*-GlcNAc in the *Drosophila* Notch EGF20 is not elongated [1]. Using glycoproteomic approach involving LC-MS/MS, *O*-GlcNAc in the 18 EGF domains in the *Drosophila* Notch EGF repeats were found to be monosaccharides in S2 cells [25]. However, in the HEK293T cells, it was suggested that the extracellular *O*-GlcNAc is further modified, at least in part, by galactose to generate *O*-GlcNAc-Gal glycan [24]. Indeed, the *O*-GlcNAc-Gal in the EGF2, EGF20, and EGF35 domains and *O*-GlcNAc-Gal-NeuAc in the EGF2 domain were observed in the mouse Notch1 EGF repeats expressed in HEK293T cells [12].

From these studies, *O*-GlcNAc structure does not appear to be conserved between insect cells and mammalian cells. In this study, we confirm the structure of *O*-GlcNAc glycan in the EGF domains on Notch1 using HEK293T cells. Our glycoproteomic analysis indicated that the *O*-GlcNAc on the Notch1 EGF repeats can be modified with galactose and sialic acid. Our results also revealed the differential *O*-GlcNAc glycosylation status among the various EGF domains. The *O*-GlcNAcylation sites in the EGF10 and EGF11 domains, known as the ligand-binding domain are poorly occupied with *O*-GlcNAc glycans, whereas *O*-GlcNAc in many other EGF domains mostly existed as monosaccharides. Thus, our glycoproteomic analysis of Notch1 EGF repeats isolated from HEK293T cells revealed structural divergence in *O*-GlcNAc glycans among the different EGF domains.

## 2. Results

### 2.1. Drosophila Notch EGF20 Expressed in HEK293T Cells is Modified with O-GlcNAc Glycan

Extracellular *O*-GlcNAc modification is not elongated and remains as a monosaccharide in the *Drosophila* Notch EGF20 (dEGF20) when expressed in S2 cells [1,25]; however, an elongated *O*-GlcNAc-Gal-Sia structure was observed in the mouse Notch1 isolated from HEK293T cells [12]. In addition to the structural differences, biological effects of *O*-GlcNAc on the Notch signal appears to be distinct in flies [3] and mammals [7]. Thus, structural difference in *O*-GlcNAc glycans might lead to functional differences of *O*-GlcNAc in Notch signaling in flies and mammals. To clarify whether the elongation of *O*-GlcNAc is dependent on cell types but not the core peptide sequence itself, we expressed dEGF20 in mammalian cells and analyzed the structure of *O*-GlcNAc glycans in dEGF20. Transiently expressed dEGF20 in HEK293T cells was purified from the culture medium and subjected to MALDI-TOF-MS analysis (Figure 1C). Three prominent peaks at *m*/*z* 8411, 8573, and 8864 were observed. As these peaks were not observed when the predicated *O*-glycosylation sites were mutated (i.e., dEGF20^ΔGlcΔFucΔGlcNAc^), the observed peaks correspond to glycoforms of dEGF20. The mass increment of 776 from the non-glycosylated EGF20 (predicted molecular weight: 7635 Da) to the first peak (*m*/*z* 8441) indicated the presence of several carbohydrate moieties. The mass increment from the first peak to the second peak (*m*/*z* 8573, +162), and that from the second to the third peak (*m*/*z* 8864, +291) indicate the presence of a hexose (Hex) and an *N*-acetylneuraminic acid (NeuAc) residues, respectively.

To confirm the modified carbohydrate moieties besides Hex and NeuAc, we generated dEGF20 variants harboring mutation at *O*-glycosylation sites (i.e., dEGF20^ΔGlcΔFucΔGlcNAc^, dEGF20^ΔGlcΔGlcNAc^, etc.) and analyzed the alternation of glycosylation patterns (Figure 1B). As expected, when *O*-Fuc and *O*-GlcNAc modification sites are removed, the three major peaks (*m*/*z* 7765, 7897, and 8029) corresponding to *O*-Glc-Xly-Xly glycans were observed (Figure 1C). In the EGF20 mutant lacking *O*-Glc and *O*-GlcNAc modification sites (dEGF20^ΔGlcΔGlcNAc^), one major peak (*m*/*z* 7735) corresponding to *O*-Fuc was observed. This was consistent with a previous report describing the diminished ability to extend the structure of *O*-Fuc in HEK293T cells [12]. In contrast, three peaks (*m*/*z* 7617, 7981, and 8273) were observed in the mutant lacking *O*-Glc and *O*-Fuc sites (dEGF20^ΔGlcΔFuc^). The increase in the mass from the first peak at *m*/*z* 7617 (corresponding to the non-glycosylated peptide) to the second peak (*m*/*z* 7982) was 365, indicating the presence of both HexNAc and Hex modification, and from the second to the third peak (*m*/*z* 8273) was 291, indicating a modification of NeuAc. These data are consistent with the view that dEGF20^ΔGlcΔFuc^ is modified with *O*-HexNAc-Hex-NeuAc glycan.

Although *O*-GlcNAc and *O*-GalNAc are indistinguishable in the MS data, the increase in the HexNAc-containing glycopeptides by EOGT-overexpression supports the conclusion that dEGF20 is indeed modified with *O*-GlcNAc. Moreover, a previous study found that reactivity of CTD110.6 antibody in detecting an *O*-GlcNAc epitope is enhanced by β-1,4-galacosidase treatment [24]. Taken together, *O*-glycan structure in *O*-GlcNAc modification site in dEGF20 is *O*-GlcNAc-Gal-NeuAc, which constitutes the glycans in the wild type dEGF20 along with *O*-Glc-Xly-Xly glycan and *O*-Fuc glycan.

### 2.2. Detection of O-GlcNAc Oligosaccharide by LC-MS/MS

To specify which EGF domains of Notch1 are modified by *O*-GlcNAc glycan in mammals, we performed glycoproteomic analysis on a secreted form of mouse Notch1 EGF repeat (mN1-EGF) transiently expressed in HEK293T cells. After separation by SDS-PAGE, mN1-EGF in the gel was digested with trypsin and analyzed using an Orbitrap Fusion mass spectrometer. To analyze the data for carbohydrate moieties of glycopeptides, specialized software for glycan analysis, namely, Byonic and GlycoPAT [26], were used. The *O*-GlcNAc glycans detected in 11 EGF domains of mN1-EGF are presented in Table 1.

**Figure 2:**
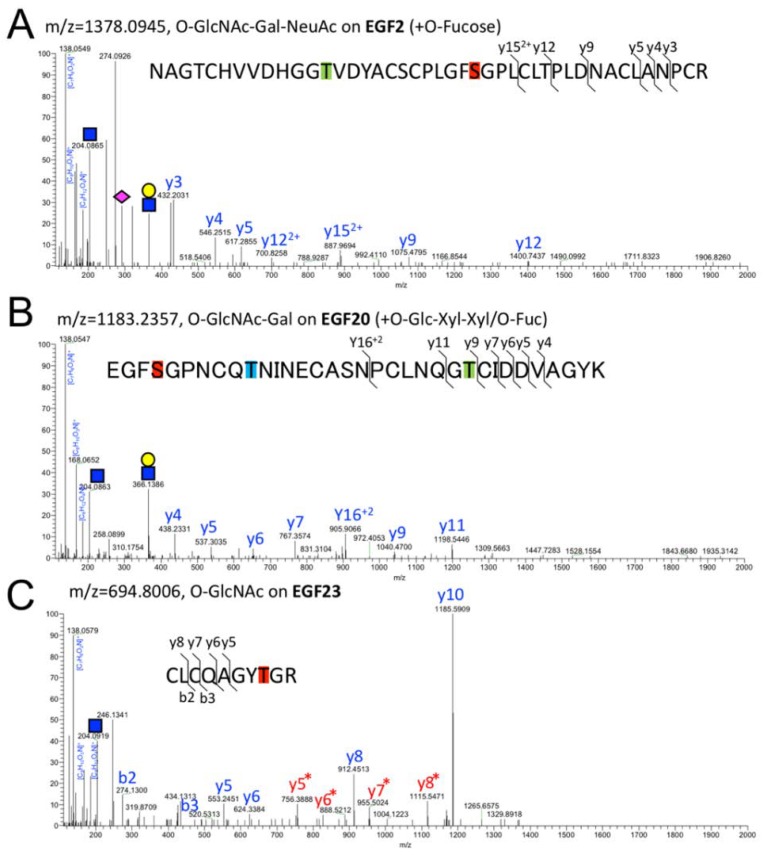
Representative MS/MS spectra for *O*-GlcNAc glycoforms. Notch1 EGF repeats expressed in HEK293T cells were isolated, digested in-gel with trypsin, and analyzed by LC-MS/MS in the HCD mode. (**A**) MS/MS spectra for the peptide with *O*-GlcNAc-Gal-NeuAc glycan from EGF2. Precursor ion corresponding to the trisaccharide glycopeptide (*m*/*z* 1378.0945) was fragmented to produce the MS/MS spectra. Neutral loss of GlcNAc (*blue square*), GlcNAc modified with Gal (*yellow circle*), and NeuAc (*purple diamond*) are indicated. The series of *y* ions were indicated in *blue colored font*. (**B**) MS/MS spectra for the peptide with *O*-GlcNAc-Gal glycan from EGF10. Precursor ion corresponding to *O*-GlcNAc-Gal glycopeptide (*m*/*z* 1183.2357) was fragmented to produce the MS/MS spectra. (**C**) MS/MS spectrum of the peptide with *O*-GlcNAc monosaccharide in EGF23. Precursor ion corresponding to *O*-GlcNAc glycopeptide (*m*/*z* 694.8006) was fragmented to produce MS/MS spectra. *O*-GlcNAcylated *y* ions are marked by an *asterisk*.

The diagnosis of the *O*-GlcNAcylated peptides was conducted by confirming the precursor ions with increase in mass by 204.0865 relative to the naked peptide and *O*-GlcNAc fragments including the *m*/*z* 204.0865 (GlcNAc), 138.0549 ([C_7_H_8_O_2_N]^+^), 168.0655 ([C_8_H_10_O_3_N]^+^), and 186.0761 ([C_8_H_12_O_4_N]^+^) in the MS/MS spectrum [27] (Figure 2A–C).

In our experimental condition, the fragment ions preserving *O*-GlcNAc moieties were not detectable with the exception of EGF23 (Figure 2C). Of note, we did not detect the peptides in other EGF domains with a modifiable residue (EGF3/7/8/9/17/19/26/29/33), probably because the trypsinized peptides were too long for detection by mass spectrometry.

*O*-GlcNAc sites that conform to the common *O*-GlcNAcylation sequence (C^5^XXX(F/Y/W)(T/S)GXXC^6^) are highlighted in *red*; *O*-Glc sites (C^1^XSX(P/A)C^2^) in blue; *O*-Fuc sites (C^2^XXXX(S/T)C^3^) in green. An atypical *O*-Glc site is highlighted in *light blue*. βOH sites (C^3^X(D/N)XXXXXXC^4^) are *underlined*. Due to the divergence in *O*-glycan structures, a subset of glycoforms containing *O*-Glc-Xyl-Xyl glycan were analyzed in EGF 15 and EGF 20. In EGF11, the presence of fragment ions corresponding to GlcNAc-Gal at *m/z* 366 discriminates between a disaccharide *O*-GlcNAc-Gal, and two monosaccharides *O*-GlcNAc and *O*-Glc.

Interestingly, the *O*-GlcNAc glycan structures identified seem to vary among the EGF domains (Table 1). Of the 11 domains analyzed, only 3 (EGF14, EGF27, and EGF28) were modified with the *O*-GlcNAc mon*O*-saccharide alone; we only observed the non-glycosylated peptides or *O*-GlcNAc glycosylated peptides. In EGF11, EGF15, EGF21, EGF23, and EGF35, besides the *O*-GlcNAc the disaccharide, *O*-GlcNAc-Gal was detected. In EGF2, EGF10, and EGF20, a trisaccharide glycoform, *O*-GlcNAc-Gal-NeuAc, was additionally observed. As shown in Figure 2A, the MS/MS spectra of glycopeptides derived from EGF2 modified with *O*-GlcNAc-Gal-NeuAc [CNCPLPYTGATCEVVLAPCATSPCK] revealed the precursor ion observed with *m*/*z* 1378.0945 and the six types of product ions with *m*/*z* 432.2031 (y3), 546.2515 (y4), 617.2855 (y5), 887.9694 (y15, 2+), 1075.4795 (y9), and 1400.641 (y12). Additionally, fragment ions derived from the glycans were produced. The *m*/*z* 204.0865 peak represents the HexNAc oxonium ion, and the *m*/*z* 138.0549, 168.0655, and 186.0761 are a series of HexNAc fragments [27]. The spectrum of *m*/*z* 366.1396 represents the HexNAc-Hex oxonium ion while the *m*/*z* 274.0926 and 292.1024 values represent the NeuAc. Similarly, representative examples of MS/MS of *O*-GlcNAc-Gal (Figure 2B, the precursor ion observed at *m*/*z* 1183.2357) in the EGF20 and *O*-GlcNAc in the EGF23 (Figure 2C, the precursor ion observed at *m*/*z* 694.8006) are shown. These results demonstrate that *O*-GlcNAc glycoforms consist of *O*-GlcNAc monosaccharide, *O*-GlcNAc-Gal disaccharide, and an *O*-GlcNAc-Gal-NeuAc trisaccharide.

### 2.3. Structural Differences in O-GlcNAc Dlycan among the EGF Domain of Mouse Notch1 EGF

**Figure 3:**
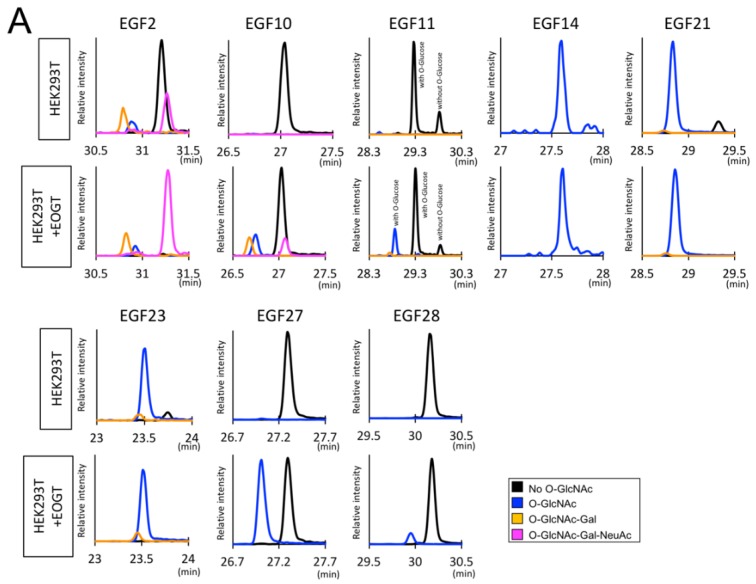

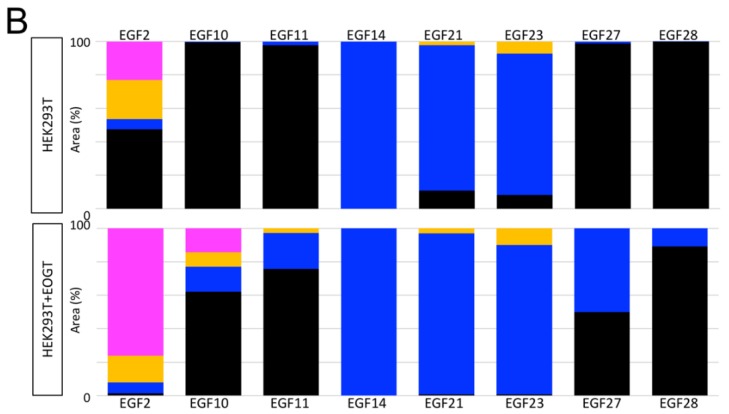
Semiquantitative analysis of *O*-GlcNAc glycans in EGF domains of Notch1. (**A**) EICs showing the relative signal intensity corresponding to the peptide with no *O*-GlcNAc (*black*), *O*-GlcNAc (*blue*), *O*-GlcNAc-Gal (*orange*), and *O*-GlcNAc-Gal-NeuAc (*purple*) in indicated EGF domains of Notch1 produced with or without exogenous EOGT expression. In EGF11, glycofroms with or without atypical *O*-Glc modification are indicated. (**B**) The peaks area of *O*-GlcNAc glycan-containing glycopeptides or un *O*-GlcNAcylated peptides are measured and expressed as percent area (%). Color code is as described in (**A**).

Based on the MS/MS spectra we noticed that the ratio of *O*-GlcNAcylated Ser/Thr over the non-*O*-GlcNAcylated Ser/Thr and the glycosylation pattern may be different among the EGF domains. To reveal the *O*-GlcNAylation stoichiometry and the glycoforms, we generated extracted ion chromatograms (EICs) in terms of non-*O*-GlcNAcylated peptides and peptides with *O*-GlcNAc, *O*-GlcNAc-Gal, or *O*-GlcNAc-Gal-NeuAc in the respective EGF domains (Figure 3A). Semi-quantitative analysis showed that EGF14, EGF21, and EGF23 were modified with *O*-GlcNAc glycans at high stoichiometries. In contrast, EGF10, EGF11, EGF27, and EGF28 had a low level of *O*-GlcNAc glycan and EGF2 had comparable levels of *O*-GlcNAc-modified peptides and non-*O*-GlcNAcylated peptides. Interestingly, the levels of *O*-GlcNAcylation in the EGF2, EGF10, EGF27, and EGF28 were increased upon EOGT overexpression. In contrast, major glycoforms observed in individual EGF domains were unchanged by exogenous EOGT with the exception of EGF2 where the ratio of trisaccharides appears to have increased. Our data from the EICs revealed differential *O*-GlcNAylation stoichiometries that were not correlated with their differential glycosylation structures observed in the mammalian Notch1 EGF repeats (Figure 3B, Table 1).

**Figure 4:**
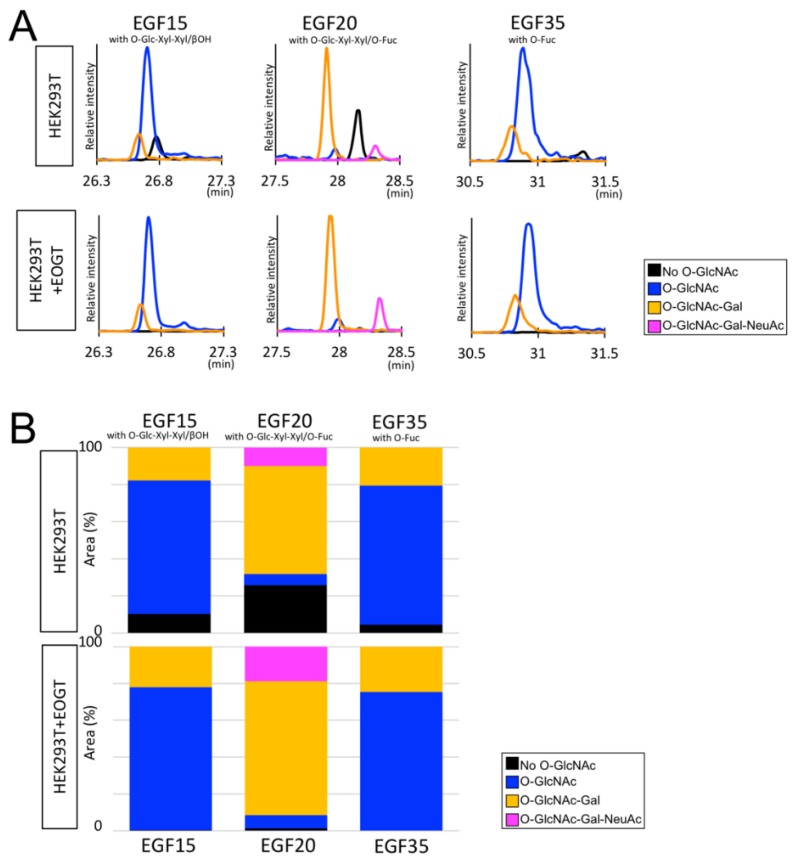
Semiquantitative analysis of *O*-GlcNAc glycans in the EGF15, EGF20, EGF35 of mouse Notch1. (**A**) EICs of EGF15, EGF20, and EGF35 modified with *O*-GlcNAc (*black line*), *O*-GlcNAc (*blue line*), *O*-GlcNAc-Gal (*orange line*), and *O*-GlcNAc-Gal-NeuAc (*purple line*) derived from mouse Notch1 with or without exogenous EOGT expression. For analysis of EGF15, EGF20, or EGF35, a subset of *O*-GlcNAc glycans containing *O*-Glc-Xyl-Xyl and βOH, *O*-Glc-Xyl-Xyl and *O*-Fuc, or *O*-Fuc are analyzed. (**B**) Relative peak area of *O*-GlcNAc glycan-containing glycopeptides or un *O*-GlcNAcylated peptides is measured and expressed as percent area (%). Color code is as described in (**A**).

One of the limitations in this study was that we could not distinguish the disaccharide (*O*-GlcNAc-Gal) or the two monosaccharides (*O*-GlcNAc and *O*-Glc) by mass spectrometry if *O*-Glc and *O*-GlcNAc were both present in the same glycopeptide (Figure 4). In case of EGF11, EICs of the *O*-GlcNAc-Gal and *O*-GlcNAc/*O*-Glc glycopeptides were clearly separated and thus successfully discriminated by the presence or absence of the HexNAc-Hex oxonium ion (*m*/*z* 366.1396). However, this is not always the case for glycopeptides harboring multiple glycoforms. To circumvent the complex situation, we analyzed the *O*-GlcNAc glycan on EGF15, EGF20, and EGF35 by focusing on the glycoforms containing the most extended form of *O*-Glc glycan, *O*-Glc-Xyl-Xyl. As with EGF2, most EGF20 were modified with the extended form of *O*-GlcNAc glycans. In contrast, as with many other domains, *O*-GlcNAc in EGF15 could be modified with Gal although the majority remained as monosaccharides. Overall, these data provide evidence that the structure and stoichiometry of *O*-GlcNAc glycans vary depending on the EGF domains within mouse Notch1.

## 3. Discussion

**Figure 5:**
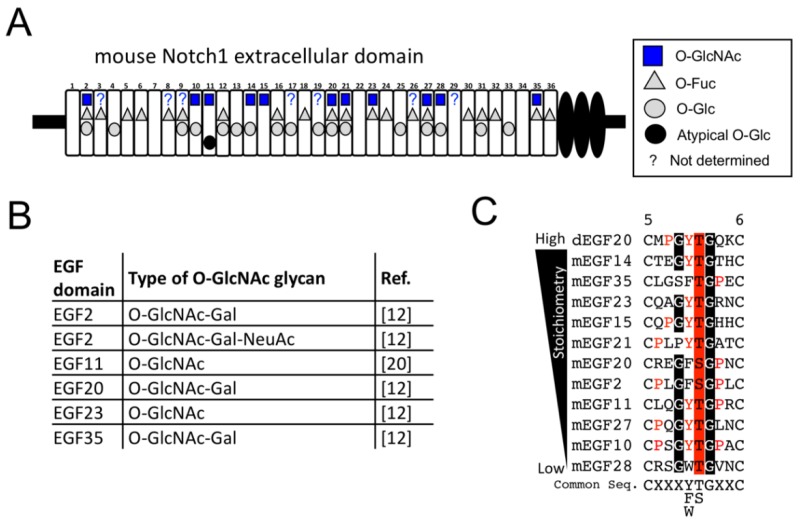
*O*-GlcNAc modification sites in the EGF repeats of mouse Notch1. (**A**) The schematic representation of the identified *O*-GlcNAcylation sites (*blue square*) found in mN1-EGF expressed in the HEK293T cells. Uncharacterized EGF domains in this study which conform to the common *O*-GlcNAcylation sequence (C^5^XXX(Y/F/W)(T/S)GXXC^6^) are shown by question marks. *O*-Glc sites (*gray circle*), atypical *O*-glucose site (*black circle*) and *O*-Fuc sites (*gray triangle*) reported by previous studies [12,25] are also marked. (**B**) The list of *O*-GlcNAcylation sites in Notch1 reported previously. (**C**) EGF domains modified with *O*-GlcNAc were listed in order of its stoichiometries. C^5^XXX(F/W/Y)(S/T)GXXC^6^ is the common sequence where *O*-GlcNAc was detected on mouse Notch1. Conserved amino acid residues are indicated by *red letters*. *O*-GlcNAcylation sites are highlighted in *red*.

Our MS spectral data provide new evidence that stoichiometries and glycoforms of *O*-GlcNAc glycan vary among the EGF domains of Notch1 (Figure 5). EGF domains with *O*-GlcNAc modification can be classified into several groups. Elongation of *O*-GlcNAc to an oligosaccharide can be observed only in EGF2, EGF10 and EGF20. However, in the majority of domains including EGF11, EGF15, EGF21, EGF23, and EGF35, only a subset of *O*-GlcNAc is modified with galactose. In the case of EGF14, EGF27, and EGF28, these domains are solely modified with *O*-GlcNAc monosaccharide, suggesting that they are refractory to further elongation. These results provide an unexpected finding in that the majority of *O*-GlcNAc moieties remain unmodified by additional sugars with an exception of a few EGF domains. Unfortunately, *O*-GlcNAcylation sites in several EGF domains failed to be analyzed by the tryptic digestion strategy. It will be important to optimize experimental conditions that enable detection of all the *O*-GlcNAc sites in Notch1 by combining multiple proteases [25]. Although this study revealed the fundamental *O*-GlcNAc glycan structures in HEK293T cell, terminal structures vary depending on the set of glycosyltransferase genes expressed in the cell. In this regard, comparative analysis using many cell types such as cancer cells and vascular endothelial cells would be important to reveal cell-type dependent modification of *O*-GlcNAc glycans in the Notch receptors.

The putative consensus sequence for *O*-GlcNAcylation of Notch1 can be proposed by comparing the modified sequences of the Notch1 EGF domains in this study. Based on the *O*-GlcNAc sites presented in Table 1, C^5^XXX(F/W/Y)(S/T)GXXC^6^ is the common sequence where *O*-GlcNAc was detected in this study. As predicted, dEGF20 harboring PYTG sequence as with Notch1 EGF15, is *O*-GlcNAcylated in HEK293T cells. However, we also noted low stoichiometries of *O*-GlcNAc in EGF10, 11, 27, and 28, and high stoichiometries in EGF14, 15, 20, 21, 23, and 35. The difference in *O*-GlcNAc stoichiometries cannot be simply explained based on the variable amino acids within the common sequence for *O*-GlcNAc modification (Figure 5C). Thus, amino acid sequence distant from the modified residue might affect physical interaction with EOGT. Further structural analysis for EOGT and substrate complex will be necessary. It is important to note that our results are somewhat inconsistent with our previous findings that alanine substitution of the *O*-GlcNAc sites at the EGF2/10/17/20 severely compromised the reactivity of *O*-GlcNAc antibody CTD110.6 [7]. One possibility is that the peptide backbone surrounding the remaining *O*-GlcNAc moieties precludes the detection by CTD110.6 antibody. Another possibility could be that CTD110.6 recognizes the cluster of *O*-GlcNAc epitopes, which is disorganized in the EGF2/10/17/20 mutant.

Our previous study on *Drosophila* EGF20 in S2 cells revealed that *O*-GlcNAc modification occurs independently of *O*-Glc and *O*-Fuc modification [1]. Current study, by using HEK293T cells, showed that *O*-GlcNAc modification occurs both in the wild type dEGF20 as well as in mutant dEGF20^ΔGlcΔFuc^. Similarly, *O*-Fuc and *O*-Glc modifications occur irrespective of the presence of other modifications. Thus, lack of *O*-glycosylation sites in dEGF20 does not apparently affect the other types of *O*-linked sugars. Unfortunately, current study failed to quantify, and thus address *O*-glycans structural extensions on dEGF20. Nonetheless, our results showed that three glycosyltransferases, Pofut1, Poglut1, and EOGT, act independently to initiate *O*-glycosylation in the EGF domains.

Current study highlights the structural differences of *O*-GlcNAc glycan in *Drosophila* and mammals. *O*-GlcNAc in *Drosophila* Notch is a mono-saccharide [1,25]. In contrast to *Drosophila*, *O*-GlcNAc is further modified with galactose and sialic acid in the EGF domains of mouse Notch1 expressed in HEK293T cells [12,24]. Our results supported the previous findings by detecting *O*-GlcNAc, *O*-GlcNAc-Gal, and *O*-GlcNAc-Gal-NeuAc in the various EGF domains. The difference in *O*-GlcNAc structures between S2 and HEK293T cells is likely due to the lack of glycosyltransferases β1,4-galactosyltrasnferases (B4galts) and sialyltransferases in *Drosophila* S2 cells. The *B4galt1* mutant in mice and CHO cells suggest their contribution in Notch signaling [28,29,30]. B4galt1 could potentially modify *O*-GlcNAc glycans besides GlcNAc in *O*-Fuc, suggesting that elongation of *O*-GlcNAc may impose additional layers of regulation. However, *O*-GlcNAc in most of EGF domains is retained as a monosaccharide. Our results raised an alternative hypothesis that arrays of *O*-GlcNAc monosaccharides may impact the structure and function of the Notch receptors.

## 4. Materials and Methods

### 4.1. Materials

pSectag2C/Eogt-IRES-EGFP, pSectag2C/dEGF20:MycHis-IRES-EGFP, and pSectag2C/mN1 EGF:MycHis-IRES-EGFP were generated as described previously [24]. pMT-Bip/EGF20:V5His, pMT-Bip/EGF20ΔGlcΔFuc:V5His, and pMT-Bip/EGF20ΔGlcΔFucΔGlcNAc:V5His were generated described previously [1].

### 4.2. Plasmid Constructs

To generate pSectag2C/dEGF20:MycHis-IRES-EGFP, pSectag2C/dEGF20ΔGlcΔFuc:MycHis-IRES-EGFP, and pSectag2C/dEGF20ΔGlcΔFucΔGlcNAc:MycHis-IRES-EGFP, dEGF20 fragments from pMT-Bip/dEGF20:V5His, pMT-Bip/dEGF20ΔGlcΔFuc:V5His, or pMT-Bip/dEGF20ΔGlcΔFuc ΔGlcNAc:V5His were excised from using *Xho*I and *Not*I and inserted in the same sites as pSectag2C/MycHis-IRES-EGFP. Other dEGF20 constructs including pSecTag2C/dEGF20ΔGlcΔGlc NAc:MycHis-IRES-EGFP and pSecTag2C /dEGF20ΔFucΔGlcNAc:MycHis-IRES-EGFP were generated using the PrimeSTAR mutagenesis basal kit (Takara, Japan). All constructs were confirmed by DNA sequencing (Supplementary Figure S1).

### 4.3. Cell Culture and Transfection

HEK293T cells were cultured in Dulbecco′s modified Eagle′s medium (DMEM) supplemented with 10% fetal bovine serum (DMEM/10% FBS). Expression vectors were transiently transfected into HEK293T cells using Lipofectamine 3000 (Invitrogen, Waltham, MA, USA) following the manufacturer′s instruction or PEI-MAX as described previously [31].

### 4.4. Sample Preparation

For the isolation of secreted MycHis tagged dEGF20 or mN1EGF, the culture media from HEK293T cells were incubated with anti-His-tag mAb-magnetic beads (MBL, Nagoya, Japan) overnight at 4 °C. The purified samples were washed extensively with PBS, and eluted with 0.1 M glycine (pH 2.7) for SDS–PAGE electrophoresis or with 0.1% TFA in 10% acetonitrile for MALDI-TOF-MS.

### 4.5. MALDI-TOF-MS Analysis

For the analysis of dEGF20 with the TOF/TOF 5800 system (AB Sciex, Framingham, MA, USA), the spectra were obtained in the linear positive ion mode using sinapinic acid (Wako, Japan) as a MALDI matrix and as described previously [1]. The purification of dEGF20 using anti-His-tag mAb-magnetic beads (MBL, Nagoya, Japan) was as described above. The samples were desalted with Ziptip (Millipore, Burlington, MA, USA) and dried in a speed vacuum system [1]. Samples were re-suspended in 1 μL of 0.1% TFA in 10% acetonitrile and spotted on the MALDI plate. After drying, 1 μL of matrix in 0.1% TFA in 10% acetonitrile was spotted on the MALDI plate and air-dried. MS spectra were recorded as the average of 2000–10,000 laser shots with laser intensity ranging from 5000–6000.

### 4.6. Hybrid Quadrupole FT Linear Ion Trap MS Analysis

The samples were subjected to SDS-PAGE using 7.5% gel and stained with a gel-negative stain kit (Nacalai, Yokohama, Japan). For in-gel digestion, gel pieces containing mN1EGF were excised and transferred to low-binding tubes. Acetonitrile was added to the tube and speed-vacuumed for 20 min for drying. The samples were reduced with 10 mM DTT in 100 mM NH_4_HCO_3_ for 30 min and alkylated with 50 mM iodoacetamide in 100 mM NH_4_HCO_3_ for 30 min. Next, the samples were incubated with 50% MeOH in 5% acetic acid at 4 °C overnight. Acetonitrile was added to the samples and dried using a speed-vacuum system. The samples were digested with 1 μg/μL trypsin gold (Promega, Madison, WI, USA) in 50 mM acetic acid for overnight at 37 °C. After desalting the solution containing digested peptides with Ziptip, each sample was dissolved within 0.1% TFA in 10% acetonitrile, and analyzed by Hybrid quadrupole FT Linear ion trap mass spectrometry using Orbitrap Fusion (Thermo, Waltham, MA, USA) coupled to an UltiMate3000 RSLCnano LC system (Dionex Co., Sunnyvale, CA, USA) with a nano-HPLC capillary column, 150 mm × 75 μm (Nikkyo Technos Co., Tokyo, Japan) via a nanoelectrospray ion source. The LC gradient with 0.1% formic acid as solution A and 0.1% formic acid 90% acetonitrile as solution B was set as follows: 5–100% B (0–45 min), 100–5% B (45–45.1 min), and 5% B (45.1–60 min). For the LC-MS/MS analysis, MS full scan (*m*/*z* 400–1600) was followed by HCD and detected by Orbitrap (orbitrap resolution: 240,000, include charge state(s): 1–4, maximum injection Time (ms): 50, collision Energy (%): 35, AGC target: 50,000).

For spectra data analysis, the raw data were searched for *O*-GlcNAc glycan-containing peptides using GlycoPAT software (MS1 tolerance: 20 ppm and MS/MS tolerance: 0.1 Da) [26]. Based on the current knowledge of *O*-glycosylation in the EGF domains, we used the glycan lists consisting of *O*-HexNAc, *O*-HexNAc-Hex, *O*-HexNAc-Hex-NeuAc, *O*-Hex, *O*-Hex-Xyl, *O*-Hex-Xyl-Xyl, and *O*-dHex. Also used was Byonic (MS1 tolerance: 10 ppm and MS/MS tolerance: 0.1 Da, Thermo, Waltham, MA, USA) as the node in Proteome Discoverer 2.2 (Thermo, Waltham, MA, USA) for *O*-glycan searches: HexNAc(1), HexNAc(1)Hex(1), HexNAc(1)Hex(1)NeuAc(1), Hex(1), Hex(1)Pen(1), Hex(1)Pen(2), dHex(1). The search parameters are as follows: fix modification, carbamidomethylation on Cys residues; variable modification, beta-hydroxylation in the Asn and Asp and methionine oxidation. For assignment of MS/MS spectra, y ions, b ions, and glycan-derived fragment ions were analyzed by Xcalibur Qual browser (Thermo, Waltham, MA, USA).

For semi-quantification, EICs were generated using Xcaliber Quan browser by selecting the most abundant isotopic peaks (monoisotopic peak or second isotope peak). After Gaussian smoothing at 5 points, the relative peak area of EICs of all observed charge states of each glycopeptide were measured. After compensation for the isotope abundance ratio, the relative amount against the total EIC peak area of all detected *O*-linked glycopeptides was calculated.

## Figures and Tables

**Table 1:** List of *O*-GlcNAcylated glycopeptides from Notch1 EGF repeats.

EGF Domain	Type of *O*-GlcNAc Glycan	Other Modification	Sequence	Precursor Ion	Charge State	RT (min)
EGF2	*O*-GlcNAc	*O*-Fuc	NAGTCHVVDHGGTVDYACSCPLGFSGPLCLTPLDNACLANPCR	1264.8098	3	30.93
EGF2	*O*-GlcNAc-Gal	*O*-Fuc	NAGTCHVVDHGGTVDYACSCPLGFSGPLCLTPLDNACLANPCR	1305.3207	3	30.82
EGF2	*O*-GlcNAc-Gal-NeuAc	*O*-Fuc	NAGTCHVVDHGGTVDYACSCPLGFSGPLCLTPLDNACLANPCR	1378.0945	3	31.27
EGF10	*O*-GlcNAc	none	AICTCPSGYTGPACSQDVDECALGANPCEHAGK	1253.5182	3	26.75
EGF10	*O*-GlcNAc-Gal	none	AICTCPSGYTGPACSQDVDECALGANPCEHAGK	980.6537	4	26.68
EGF10	*O*-GlcNAc-Gal-NeuAc	none	AICTCPSGYTGPACSQDVDECALGANPCEHAGK	1053.4281	4	27.06
EGF11	*O*-GlcNAc	*O*-Glc βOH	CLNTLGS FECQCLQGYTGPR	915.3972	3	28.79
EGF11	*O*-GlcNAc-Gal	*O*-Glc βOH	CLNTLGS FECQCLQGYTGPR	969.0807	3	28.67
EGF14	*O*-GlcNAc	βOH	CLDGPNTYTCVCTEGYTGTHCEVDIDECDPDPCHYGSCK	1216.8245	4	27.61
EGF15	*O*-GlcNAc	*O*-Glc-Xyl βOH	DGVATFTCLCQPGYT GHHCETNINECHSQPCR	1081.1904	4	26.71
EGF15	*O*-GlcNAc	*O*-Glc-Xyl-Xyl βOH	DGVATFTCLCQPGYT GHHCETNINECHSQPCR	1114.2009	4	26.70
EGF15	*O*-GlcNAc-Gal	*O*-Glc-Xyl-Xyl βOH	DGVATFTCLCQPGYTGHHCETNINECHSQPCR	1154.9644	4	26.64
EGF20	*O*-GlcNAc	*O*-Glc-Xyl-Xyl *O*-Fuc	EGFSGPNCQTNINECAS NPCLNQGT CIDDVAGYK	1142.7227	4	27.99
EGF20	*O*-GlcNAc-Gal	*O*-Glc-Xyl-Xyl *O*-Fuc	EGFSGPNCQTNINECAS NPCLNQGT CIDDVAGYK	1183.2357	4	27.92
EGF20	*O*-GlcNAc-Gal-NeuAc	*O*-Glc-Xyl-Xyl *O*-Fuc	EGFSGPNCQTNINECAS NPCLNQGT CIDDVAGYK	1256.0098	4	28.32
EGF21	*O*-GlcNAc	none	CNCPLPYTGATCEVVLAPCATSPCK	1010.7797	3	28.85
EGF21	*O*-GlcNAc-Gal	none	CNCPLPYTGATCEVVLAPCATSPCK	1064.797	3	28.77
EGF23	*O*-GlcNAc	none	CLCQAGYTGR	694.8006	2	23.51
EGF23	*O*-GlcNAc-Gal	none	CLCQAGYTGR	775.8269	2	23.46
EGF27	*O*-GlcNAc	none	CTCPQGYTGLNCQNLVR	749.0013	3	27.01
EGF28	*O*-GlcNAc	none	SGWTGVNCDVLSVSCEVAAQK	824.0469	3	29.95
EGF35	*O*-GlcNAc	*O*-Fuc	SPTCLCLGSFTGPECQFPASSPCVGSNPCYNQGT CEPTSENPFYR	1368.322	4	30.93
EGF35	*O*-GlcNAc-Gal	*O*-Fuc	SPTCLCLGSFTGPECQFPASSPCVGSNPCYNQGT CEPTSENPFYR	1408.8363	4	30.82

## Supplementary Materials

Supplementary materials are available online. Figure S1: DNA sequence of pSectag2C/dEGF20:MycHis-IRES-EGFP series.
