# Supplementary material for: Structural Divergence in O-GlcNAc Glycans Displayed on Epidermal Growth Factor-like Repeats of Mammalian Notch1
Source: Molecules. 2018 Jul 17;23(7):1745. doi: 10.3390/molecules23071745 (PMC6099671; doi:10.3390/molecules23071745)
Supplement: Supplementary file 1 [file molecules-23-01745-s001.pdf]

pSecTag2C/EGF20-MycHis/IRES-EGFP
(atagacgagtgcagcagcaatccctgccagcatggtggcacctgttatgataagctcaacgccttcagttgccaatgcatgccgggctatacgggccaaaagtgcgaaacgaat)

pSecTag2C/EGF20ΔGlcΔFuc-MycHis/IRES-EGFP
(atagacgagtgcagc**gcc**aatccctgccagcatggtggc**gtc**tgttatgataagctcaacgccttcagttgccaatgcatgccgggctatacgggccaaaagtgcgaaacgaat)

pSecTag2C/EGF20ΔGlcΔFucΔGlcNAc-MycHis/IRES-EGFP
(atagacgagtgcagc**gcc**aatccctgccagcatggtggc**gtc**tgttatgataagctcaacgccttcagttgccaatgcatgccgggctat**gcg**ggccaaaagtgcgaaacgaat)

pSecTag2C/dEGF20ΔGlcΔGlcNAc-MycHis/IRES-EGFP
(atagacgagtgcagc**gcc**aatccctgccagcatggtggcacctgttatgataagctcaacgccttcagttgccaatgcatgccgggctat**gcg**ggccaaaagtgcgaaacgaat)

pSecTag2C/dEGF20ΔFucΔGlcNAc-MycHis/IRES-EGFP
(atagacgagtgcagcagcaatccctgccagcatggtggc**gtc**tgttatgataagctcaacgccttcagttgccaatgcatgccgggctat**gcg**ggccaaaagtgcgaaacgaat)

Figure S1: DNA sequence of pSectag2C/dEGF20:MycHis-IRES-EGFP series.
